# The crosstalk between microRNAs and the Wnt/β-catenin signaling pathway in cancer

**DOI:** 10.18632/oncotarget.12923

**Published:** 2016-10-26

**Authors:** Yin Peng, Xiaojing Zhang, Xianling Feng, Xinmim Fan, Zhe Jin

**Affiliations:** ^1^ Department of Pathology, The Shenzhen University School of Medicine, Shenzhen, Guangdong, People's Republic of China; ^2^ Shenzhen Key Laboratory of Micromolecule Innovatal Drugs, The Shenzhen University School of Medicine, Shenzhen, Guangdong, People's Republic of China; ^3^ Shenzhen Key Laboratory of Translational Medicine in Tumors, The Shenzhen University School of Medicine, Shenzhen, Guangdong, People's Republic of China; ^4^ Department of Pathology, Wuhan University School of Basic Medical Sciences, Hubei, People's Republic of China

**Keywords:** microRNA, Wnt/β-catenin signaling pathway, cancer

## Abstract

Mounting evidence has indicated microRNA (miR) dysregulation and the Wnt/β-catenin signaling pathway jointly drive carcinogenesis, cancer metastasis, and drug-resistance. The current review will focus on the role of the crosstalk between miRs and the Wnt/β-catenin signaling pathway in cancer development. MiRs were found to activate or inhibit the canonical Wnt pathway at various steps. On the other hand, Wnt activation increases expression of miR by directly binding to its promoter and activating transcription. Moreover, there are mutual feedback loops between some miRs and the Wnt/β-catenin signaling pathway. Clinical trials of miR-based therapeutic agents are investigated for solid and hematological tumors, however, challenges concerning low bioavailability and possible side effects must be overcome before the final clinical application. This review will describe current understanding of miR crosstalk with the Wnt/β-catenin signaling cascade. Better understanding of the regulatory network will provide insight into miR-based therapeutic development.

## INTRODUCTION

MiR, as an evolutionarily conserved gene expression regulator, participates in many fundamental physiological processes. In cancer development, miR functions as a tumor suppressor or an oncogene by targeting specific genes in a 3′UTR dependent manner. This mechanism occurs in a variety of tumors. It is well known that Wnt/β-catenin drives the carcinogenesis, cancer progression, and metastasis in many tumors. Emerging evidence has indicated the importance of interaction between the Wnt/β-catenin signaling pathway (Wnt pathway for short) and miR mediated gene regulation in cancer development. In this paper, we would like to summarize current understanding of the crosstalk between miRs and the Wnt pathway with respect to oncogenesis, cancer metastasis, and drug-resistance.

### MiRs overview

MiRs are short (18-25 nucleotides) non-coding RNAs. They repress gene expression through interaction with the 3′UTR of target genes, either inhibiting mRNA translation and/or promoting mRNA degradation. One miR family is potentially able to target 500 genes on average [1]. Likewise, approximately 60% of the mRNAs can be targeted by one or multiple miRs [1]. For example, miR-34 was identified as targeting a number of genes including WNT1/3, β-catenin, LRP6, LEF1, AXIN2, TCF7 post-transcriptionally [2]. MiR-145, -133a and -133b were found to target gene FSCN1 to inhibit cell proliferation and invasion [3]. Thus, changing the expression of miRs will impact a cascade of modifications in gene expression.

### MiRs in cancer

Genome-wide profiling has shown that miRs are frequently aberrantly expressed in human neoplasms including gastric cancer, hepatocellular carcinoma (HCC), breast cancer, glioblastoma, prostate cancer and colorectal cancer (CRC) [2]. Software prediction in combination with experimental validation showed that miRs are involved in tumorigenesis, angiogenesis, metastasis, and chemo-resistance by directly targeting specific oncogenes or tumor suppressors. For example, let-7c was shown to be down-regulated in gastric cancer, which was related to Helicobacter pylori-induced gastric carcinogenesis [4]. MiR-194 was identified to promote angiogenesis by suppressing the endogenous angiogenesis inhibitor thrombospondin1 in colon cancer [5]. MiR-21, one of the most well studied miRs, was found to be overexpressed in a wide variety of tumors including glioblastoma, colon, breast, and gastric cancers, leading to enhanced cell invasion and metastasis [6]. In addition, miRs can be used as diagnostic, therapeutic, and prognostic factors. Based on a meta-analysis, the circulating miR-21 expression is a useful noninvasive biological marker for the earlier detection of digestive system cancers such as CRC, HCC, gastric cancer, and a promising prognostic marker for digestive system cancers in the Asian population [7].

### Canonical Wnt pathway overview

The Wnt pathway is critical for embryo development and adult tissue homeostasis. Without Wnt protein stimulation, β-catenin is anchored by a destruction complex comprised of APC, GSK3β and Axin [8]. β-catenin is phosphorylated by CK1α and GSK3β, followed by ubiquitination by β-TRCP, resulting in β-catenin proteasomal degradation. The activation of the pathway initiates from the Wnt proteins binding to the receptor Frizzled (FZD) and co-receptor LDL receptor-related protein (LRP) 5 or LRP6. Ligand binding to the receptor leads to the phosphorylation of Dishevelled (DVL). Phosphorylated DVL recruits AXIN and GSK3β to the cell membrane, thereby, inhibiting the phosphorylation of β-catenin and allowing β-catenin to dissociate from the destruction complex. Next, the β-catenin that accumulates localizes to the nucleus and interacts with the TCF/LEF to transactivate downstream gene expression including CyclinD, c-Myc, CD44. Wnt signaling can be suppressed by antagonist molecules such as Wnt inhibitory factor 1 (WIF1), secreted frizzled-related protein family (sFRP) and Dickkopf (DKK) (reviewed in [9]).

### Canonical Wnt pathway in cancer

Aberrant activation of the Wnt pathway is highly associated with tumorigenesis and tumor metastasis. Wnt signaling components are commonly mutated in many human cancers. Exome-sequencing has revealed that most of CRC patients harbor loss of function mutations in APC [10]. In some CRC patients, Axin2 is mutated [11], or β-catenin has activating point mutations [12]. A rare but recurrent fusion between VTI1A and TCF7L2, the gene encoding TCF4, has also been found in CRC patients [13]. In CRC and a host of other cancers, Wnt signaling components are mutated. Oncogenic β-catenin mutations are present in melanoma, HCC, gastric, pancreas, ovarian, and endometrial cancers (reviewed in [14]). Inactivating mutations of Axin were observed in HCC [15]. In addition to germline/somatic mutations, aberrant activation of Wnt pathway can result from epigenetic modifications, such as miR regulation. We will discuss this in detail in later sections.

## CROSSTALK BETWEEN MIR AND THE WNT PATHWAY

**Figure 1:**
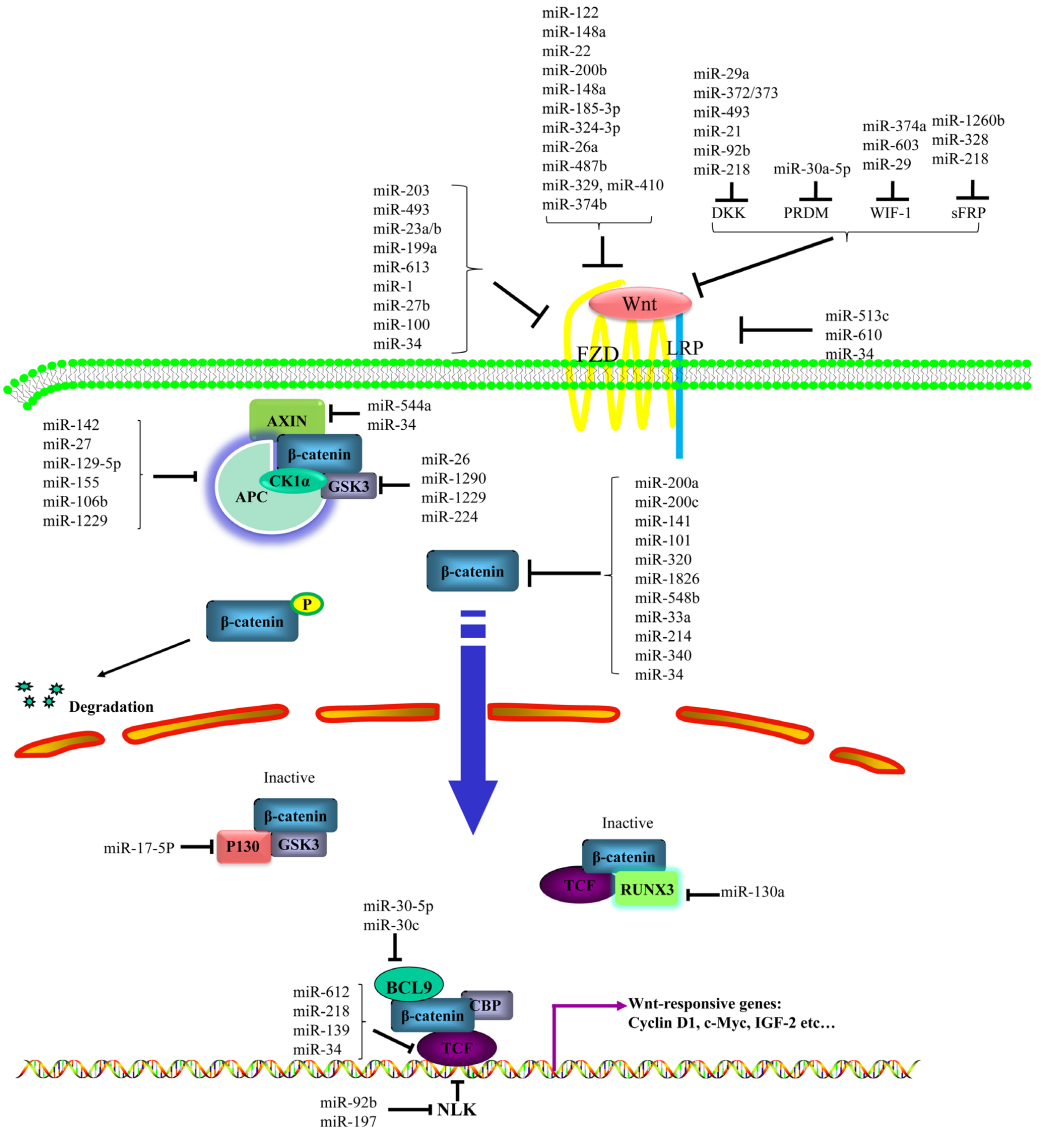
Regulation of miRs on Wnt/β-catenin signaling pathway MiRs activate or inhibit canonical Wnt pathway at multiple levels by targeting Wnt ligands/receptors and ligands/receptors associated proteins, β-catenin, β-catenin interacting complex, Wnt pathway transcription factors, multiple Wnt signaling pathway components and components in other signaling pathways.

Dysregulation of miR induces constitutively active Wnt signaling activity in cancer, while expression of miR, in turn, is intensely controlled by Wnt signaling. In order to identify miRs that regulate the Wnt pathway activity, 470 miRs were screened in a cell-based assay in human HEK293 cells and 38 candidate miRs were identified [16]. MiRs activate or repress the Wnt pathway at multiple levels by targeting Wnt ligand/receptor and ligand/receptor associated proteins, β-catenin, β-catenin interacting complex, Wnt pathway transcription factors, multiple Wnt signaling pathway components and components in other signaling pathways (Figure 1). Meanwhile, Wnt activation increases expression of miR through binding of β-catenin to TCF/LEF which then binds to promoter regions to activate transcription. Moreover, there are mutual feedback loops between some miRs and Wnt signaling components. In summary, Wnt activation and miR mediated gene regulation are reciprocal causation to drive oncogenesis.

### MiRs modulate the Wnt pathway

#### Targeting Wnt ligands/receptors and ligands/receptors associated inhibitory proteins

It is well established that Wnt activation initiates from the Wnt proteins binding to the FZD and LRP5 or LRP6. If miRs target the Wnt ligand/ receptor such as WNT, FZD and LRP, the pathway is repressed. Both miR-122 and -148a were found to be down-regulated in HCC and to repress the Wnt pathway by targeting WNT1. MiR-122 was shown to suppress cell proliferation and promote cell apoptosis [17]. MiR-148a was reported to inhibit the metastasis of HCCs by blocking EMT and cancer stem cell like properties [18]. Other miRs including miR-22, -200b, -185-3p, -324-3p, -26a, 487b, -329, -410 and -374b also target Wnt ligand to inhibit the Wnt/β-catenin signaling pathway [19–26] (Figure 1 and Table 1). Another group of miRs targeting FZD and LRP act like tumor suppressors. For example, miR-1 and -100 were identified to suppress breast cancer progression by inhibiting the Wnt pathway through targeting the FZD7 and FZD8 respectively [27, 28]. MiR-513c was found to suppress glioblastoma cell proliferation predominantly through direct suppression of the expression of LRP6 [29].

**Table 1:** MiRs targeting Wnt ligands/receptors and associated proteins

miR	Target in Wnt signaling	Effect	Cell Type	Reference
***Targeting Wnt ligands/receptors and associated proteins***				
miR-122	WNT1	Tumor suppressor	HCC, Glioma	[17, 114]
miR-148a	WNT1,WNT10B	Tumor suppressor	HCC, Endometrial cancer	[18, 116]
miR-22	WNT1	Tumor suppressor	Gastric cancer	[19]
miR-200b	WNT1, PKCα, ZEB2	Tumor suppressor	Gastric cancer, Glimoma	[19, 20, 46]
miR-185-3p	WNT2B	Tumor suppressor	Nasopharyngeal carcinoma	[21]
miR-324-3p	WNT2B	Tumor suppressor	Nasopharyngeal carcinoma	[22]
miR-26a	WNT5A	Tumor suppressor	Prostate cancer	[25]
miR-487b	WNT5A, SUZ12, BMI1, MYC, and KRAS	Tumor suppressor	Lung cancer	[23]
miR-329, miR-410	WNT7B	Tumor suppressor	Oral squamous cell carcinoma	[24]
miR-374b	WNT16, AKT1	Tumor suppressor	T-cell Lymphoblastic Lymphoma	[26]
miR-203	FZD2	Tumor suppressor	Lung cancer	[117]
miR-493	FZD4	Tumor suppressor	Bladder cancer	[118]
miR-23a/b	FZD5, FZD7	Tumor suppressor	Pancreatic cancer, Colon cancer	[119, 120]
miR-199a	FZD7, E-cadherin	Tumor suppressor/ Oncogene	HCC, Gastric cancer	[121, 122]
miR-613	FZD7	Tumor suppressor	Prostate cancer	[123]
miR-1	FZD7, TNKS2	Tumor suppressor	Breast cancer	[27]
miR-27b	FZD7	Tumor suppressor	Gastric cancer	[124]
miR-100	FZD8	Tumor suppressor	Breast cancer	[28]
miR-513c	LRP6	Tumor suppressor	Glioblastoma	[29]
miR-610	LRP6, TBL1X	Tumor suppressor	HCC	[125]
miR-218	LGR4	Tumor suppressor	Prostate cancer	[126]
miR-374a	WIF1, PTEN, WNT5A	Oncogene	Breast cancer	[30, 31]
miR-603	WIF1, CTNNBIP1	Oncogene	Glioma	[32]
miR-29	demethylation of WIF1, N-myc interactor	Tumor suppressor/ Oncogene	NSCLC, Breast cancer	[127, 128]
miR-1260b	sFRP1, DKK2, Smad4	Oncogene	Renal cancer	[33]
miR-328	sFRP1	Oncogene	Glioma	[34]
miR-29a	DKK1, Kremen2, sFRP2	Oncogene	Pancreatic cancer	[35]
miR-372/373	DKK1	Oncogene	CRC, Breast Cancer	[36]
miR-493	DKK1	Oncogene	Gastric cancer	[129]
miR-21	DKK2, TGFβR2	Oncogene	Oral cancer, Colon cancer	[37, 130]
miR-92b	DKK3,NLK	Oncogene	Glioma	[38, 74]
miR-218	SOST, DKK2, SFRP2	Oncogene	Breast cancer	[112]
miR-30a-5p	PRDM	Oncogene	Glioma	[131]

Conversely, Wnt signaling is activated when miRs target Wnt antagonists such as WIF, sFRP, DKK, and PR domain proteins (PRDM). MiR-374a [30, 31], -603 [32] -1260b [33], -328 [34] were discovered to activate Wnt/β-catenin signaling by targeting antagonists WIF1 or sFRP1. The DKK and PRDM protein families block Wnt signaling by binding to the LRP5/LRP6. MiR-29a [35], -372&373 [36], -21 [37] and -92b [38] were found to enhance the Wnt pathway activity *via* targeting DKK, which resulted in either accelerating cancer cell proliferation/invasion or inducing resistance to chemotherapy. Additional examples of miR regulating Wnt ligands/receptors and their associated inhibitory proteins are listed in Table 1 and Figure 1.

The Wnt receptor is at the crux between extracellular ligands and intracellular responses such as survival or apoptosis, proliferation or growth arrest, drug susceptibility or drug resistance. Thus targeting the receptor and its associated proteins by miRs fundamentally influences signaling transduction at the initiation phase. As discussed, miR regulation of the Wnt/β-catenin cascade is multi-faceted with regards to receptor activation and warrants interest for possible therapeutic interventions.

#### Targeting β-catenin

β-catenin, the most important signaling factor in the Wnt pathway, transduces the signal by directly binding to TCF/LEF transcription factors and turning on downstream gene expressions which promote cell proliferation, migration, EMT, cancer cell metastasis and chemo-resistance [9]. A number of miRs have been found to suppress the Wnt pathway by modulating β-catenin. The well-studied miR-200 cluster is usually under-expressed in cancer cells and is identified to target β-catenin to inhibit cancer progression [39–45]. Five members are included in the family: miR-200a, -200b, -200c, -141 and -429. MiR-200a was identified to be reduced in meningioma, HCC, and gastric cancer [39–43]. This decrease blocked the Wnt pathway by two mechanisms: it targeted the 3′-UTR of β-catenin and the EMT transcription factors zinc finger E-box binding homeobox 1/2 (ZEB1/2). The resulting effect was the reduction of β-catenin and inhibition of Wnt signaling. MiR-200c was shown to repress Wnt pathway by targeting β-catenin directly in breast cancer [44]. MiR-200b was established to have suppressive effects on the proliferation, migration, invasion and EMT of glioma cells *via* targeting ZEB2 [46]. Moreover, miR-141 was also found to suppress β-catenin expression in breast cancer [45] and down-regulate SOX17 expression, causing activation of Wnt signaling in esophageal cancer [47].

Many other miRs target β-catenin as well, besides the miR-200 family. MiR-214 [48, 49], -320 [50], -101 [51], -1826 [52, 53], -548b [30], and -33a [54], were found to block Wnt pathway by targeting the 3′UTR of β-catenin in HCC, prostate, breast, colon, urological, and lung cancer, respectively (Figure 1 and Table 2).

**Table 2:** MiRs targeting β-catenin/TCF and associated proteins

miR	Target in Wnt signaling	Effect	Cell Type	Reference
***Targeting β-catenin***				
miR-200a	β-catenin, ZEB1/2	Tumor suppressor	Meningiomas, Gastric Cancer, HCC	[39–43]
miR-200c	β-catenin	Tumor suppressor	Breast cancer	[44]
miR-141	β-catenin, SOX17	Tumor suppressor	Breast cancer, Esophageal cancer	[45, 47]
miR-101	β-catenin	Tumor suppressor	Colon cancer	[51]
miR-320	β-catenin,FOXM1	Tumor suppressor	Prostate cancer, Colon cancer	[50, 132]
miR-1826	β-catenin	Tumor suppressor	Bladder cancer, Renal cancer	[52, 53]
miR-548b	β-catenin	Tumor suppressor	Lung cancer	[30]
miR-33a	β-catenin	Tumor suppressor	HCC	[54]
miR-214	β-catenin, EZH2	Tumor suppressor	HCC	[48, 49]
miR-340	β-catenin, ROCK1, c-MYC	Tumor suppressor	Breast cancer	[133]
***Targeting multiple Wnt signaling components***				
miR-34	WNT1/3, β-catenin, LRP6, LEF1, AXIN2,TCF7	Tumor suppressor	Lung cancer, Breast cancer, CRC, Prostate cancer, HCC	[55, 57–59, 119, 134]
***Targeting β-catenin interacting proteins***				
miR-142	APC	Oncogene	Breast cancer	[135]
miR-27	APC	Oncogene	Gastric cancer	[136]
miR-129-5p	APC	Oncogene	Squamous cell carcinoma	[137]
miR-155	APC, CK1α, HBP1	Oncogene	HCC, Papillary thyroid carcinoma, Liposarcoma, Glioma	[61, 62, 138, 139]
miR-106b	APC	Oncogene	HCC	[140]
miR-1229	APC, GSK3β	Oncogene	Breast cancer	[141]
miR-26	GSK3β	Oncogene	Cholangiocarcinoma	[63]
miR-1290	GSK3β	Oncogene	Lung adenocarcinoma	[64]
miR-224	GSK3β, SFRP2	Oncogene	CRC	[142]
miR-146a	Numb	Oncogene	CRC	[113]
miR-544a	E-cadherin, AXIN2	Oncogene	Gastric cancer	[65]
miR-145	Catenin δ-1	Tumor suppressor	Colon cancer	[143]
miR-490-3p	FRAT1	Tumor suppressor	CRC	[144]
***Targeting Wnt signaling related transcription factors***				
miR-612	TCF/LEF	Tumor suppressor	HCC	[145]
miR-218	LEF1, BMI1	Tumor suppressor	Glioblastoma, Glioma	[66, 146]
miR-139	TCF-4	Tumor suppressor	HCC	[67]
miR-17-5P	P130, HBP1	Oncogene	CRC, Breast cancer	[69, 70]
miR-197	NLK	Oncogene	Ovarian cancer	[75]
miR-130a	RUNX3	Oncogene	HCC, Gastric Cancer	[72, 147]
miR-30-5p/ miR-30c	BCL9	Tumor suppressor	Myeloma, Prostate cancer	[148, 149]
miR-452	Sox7	Oncogene	HCC	[150]

Certain miRs not only target β-catenin alone, but also directly suppress a set of multiple components of the Wnt signaling cascade to regulate Wnt activity such as miR-34. MiR-34 trans-activated by p53 inhibits the Wnt pathway by targeting the 3′-UTRs of a set of conserved targets which are elements of the Wnt pathway. These genes include WNT1/3, β-catenin, LRP6, AXIN2 and LEF1 [55–59]. The inactivating mutation of p53 turns on canonical Wnt signaling and enhances EMT by inducing miR-34 mediated reduction of target gene expression in human cancer.

β-catenin is the central and most well studied signaling molecule in the Wnt pathway. By targeting β-catenin, miRs directly repress the Wnt signaling activity (Figure 1 and Table 2). Moreover, miR-34s are able to target a number of Wnt signaling components, allowing efficient regulation of important physiological functions associated with cancer initiation and progression. This is a very appealing property for therapy development. As a result, a miR mimic targeting miR-34 was developed into a therapeutic (MRX34) and is in a phase 1 clinical trial for solid and hematological tumors [60].

### Targeting β-catenin-interacting complex

Due to the significance of β-catenin in Wnt signaling, miRs targeting the β-catenin interacting proteins such as APC, GSKβ, AXIN and similar associates, consequently regulate Wnt signaling. APC is a scaffolding protein for the APC/GSK3β/CK1α/β-catenin/AXIN complex which mediates destruction of β-catenin. It is a common tumor suppressor, mutated in a variety of cancers. MiR-155 has been reported to negatively regulate APC in multiple carcinomas including HCC and papillary thyroid carcinoma [61, 62] to promote cell proliferation and tumorigenesis. These data suggest miR-155 as a promising target for diagnosis and treatment of HCC and papillary thyroid cancer.

In regard to the kinase in the “destruction complex”, GSK-3β was targeted by miR-26a as revealed in target prediction and validated in biological experiments. MiR-26a targets GSK-3β to promote cholangiocarcinoma growth through concomitant activation of Wnt/β-catenin [63]. GSK-3β is also a target of miR-1290 in lung cancer [64].

Another scaffolding protein in the “destruction complex”, AXIN2 is targeted by miR-544a. MiR-544a was identified as an EMT-inducing miR in a cell-based reporter system with a 328-miRs library. In gastric cancer, it targets E-cadherin and AXIN2 to promote β-catenin translocation to the nucleus, resulting in active Wnt signaling [65]. It suggests that miR-544a might be a potential target for treating advanced gastric cancer. Additional miRs targeting β-catenin associated factors are listed in Table 2 and Figure 1.

In summary, miRs regulate the Wnt pathway by targeting β-catenin directly and by inhibiting expression of β-catenin interacting proteins, revealing a regulatory network that is intertwined and complicated.

### Targeting Wnt pathway transcription factors

The established action mechanism of β-catenin is activation of downstream gene expression by binding the transcriptional factor TCF/LEF. MiRs that target the TCF/LEF transcription factors and other associated co-activators/co-repressors regulate transcription transduced by Wnt signaling (Figure 1 and Table 2). For example, miR-218 inhibits glioblastoma cell invasiveness by suppressing the Wnt pathway *via* direct targeting of oncogenic transcription factor LEF1 [66]. MiR-139 attenuates the proliferative and invasive ability of HCC by inhibiting the Wnt pathway through TCF4 [67].

Other transcriptional factors and co-activators/co-repressors besides the TCF family are modulated by miRs to affect Wnt signaling. P130 is a transcription factor associated with GSK3β which sequesters β-catenin in an inactive form as a P130/GSK3β/β-catenin complex in the cell nucleus [68]. It was found that P130 is targeted by miR-17-5p to activate the Wnt pathway in CRC to promote cancer advancement [69]. HBP1, a transcriptional repressor for the Wnt pathway, is also targeted by miR-17-5p to activate Wnt signaling to promote cell migration and invasion in breast cancer [70]. RUNX3 (Runt related transcription factor 3) forms a ternary complex with β-catenin/TCF4 to inhibit Wnt signaling activity [71]. Elevated expression of miR-130a may directly repress RUNX3 to activate Wnt/β-catenin signaling and subsequently lead to chemo-resistance in HCC cells [72].

NLK (Nemo-like kinase), an evolutionarily conserved protein kinase, is an inhibitor for the Wnt pathway by binding to and phosphorylating TCF/LEF-1 family proteins [73]. MiR-92b was identified to promote glioma proliferation and invasion by targeting NLK which resulted in active Wnt/β-catenin signaling [74]. More recently, miR-197 was reported to activate Wnt signaling by downregulation of NLK in ovarian cancer [75].

Collectively, research has documented the involvement of miRs in regulating the Wnt pathway at the last checkpoint-mRNA expression. Transcriptional regulation was observed by direct control of TCF/LEF expression and by inhibition of the formation of the active TCF/β-catenin complex. Thus, miR regulation of transcription adds another layer of fine tuning the signaling cascade.

### Targeting components in other signaling pathways

More recently, the crosstalk between Wnt signaling and other signaling pathways has been described in more detail. A great number of miRs have been found to regulate the Wnt signaling pathway indirectly by targeting elements in other pathways (Table 3). The function of components from other signaling pathways varied from one to another. They can be classified into three major groups. The largest group comprises transcriptional factors such as E2F1 [76], smad [77] and ZEB [78]. The next set is the group of enzymes including Phosphatase and tensin homolog (PTEN) [79], and Zinc and Ring Finger 3 (ZNRF3) [80]. The final group involves the receptor associated proteins such as Roundabout 3 (ROBO3) [81] and Solute Carrier Family 34 Member 2 ( SLC34A2) [82].

**Table 3:** MiRs targeting other signaling pathway components

miR	Target in Wnt signaling	Effect	Cell Type	Reference
***Targeting other signaling pathway components***				
Targeting Transcription factors				
miR-182-5p	Smad4, RECK	Oncogene	Bladder cancer	[77]
miR-93	Smad7, ZNRF3	Tumor suppressor/ Oncogene	CRC, Lung cancer	[87, 95]
miR-33b	ZEB1	Tumor suppressor	Lung adenocarcinoma	[78]
miR-376c	LRH-1	Tumor suppressor	NSCLC	[151]
miR-145	Oct4	Tumor suppressor	Lung cancer	[152]
miR-191	WT1	Oncogene	Lung cancer	[153]
miR-153	WWOX	Oncogene	HCC	[154]
miR-19b/20a/92a	E2F1, HIPK1	Oncogene	Gastric cancer	[84]
miR-19	MEF2D	Tumor suppressor	Gastric cancer	[86]
Targeting Enzyme				
miR-301a	PTEN	Oncogene	Breast cancer	[79]
miR-429	PTEN	Oncogene	HCC	[88]
miR-146b-5p	ZNRF3	Oncogene	Thyroid cancer, Osteosarcoma	[80, 155]
miR-506	EZH2	Tumor suppressor	Colon cancer	[92]
miR-144	EZH2	Tumor suppressor	Bladder cancer	[93]
miR-29c	GNA13, PTP4A	Tumor suppressor	CRC	[156]
Targeting Receptor associated proteins				
miR-576-5p	ITGBL1	Oncogene	NSCLC	[157]
miR-410	SLC34A2	Oncogene	NSCLC	[82]
miR-483-5p	RhoGDI1, ALCAM	Oncogene	Lung adenocarcinoma	[158]
miR-494	CXCR4	Tumor suppressor	Breast cancer	[159]
miR-152	TNFRSF6B	Tumor suppressor	HCC	[160]
miR-383	ROBO3	Tumor suppressor	Pancreatic cancer	[81]

#### Targeting transcription factors in other signaling pathways

Transcription factor E2F1 preferentially binds to retinoblastoma protein pRB to prevent cell-cycle progression. It mediates p53-dependent/independent apoptosis. E2F1 was shown to suppress the Wnt pathway by trans-activating Catenin Beta Interacting Protein 1 (CTNNBIP1) [76] or by up-regulating AXIN2 [83]. MiR-19b/20a/92a was found to sustain the stem cell self-renewal and promote cell proliferation in gastric cancer by increasing Wnt signaling through reduction of E2F1 and dishevelled-associated protein1 (HIPK1) expression [84].

Myocyte enhancer factor 2D (MEF2D) is a transcription factor of the MEF2 family. The family members were found to be involved in carcinogenesis and cancer progression [85]. MiR-19 inhibits cell proliferation in gastric cancer by targeting MEF2D. MEF2D inhibition leads to repression of the Wnt pathway. The miR-19/MEF2D/Wnt/β-catenin axis is critical for gastric cancer cell survival and proliferation, suggesting miR-19 as a potential therapeutic target for gastric cancer [86].

Take another transcription factor for example, smad4 is an important transcription factor for TGFβ signaling which participates in multiple biological processes including cell survival, apoptosis, growth and differentiation. For the TGFβ/Smad pathway, smad4, smad7 and TGFβ are targeted by miR-182-5p [77], miR-93 [87] and miR-21 [37] in bladder cancer, CRC, and colon cancer respectively.

#### *Targeting enzymes in other signaling pathway*s

In addition to transcription factors, miR regulates Wnt signaling indirectly by targeting enzymes in other signaling pathways. PTEN, a critical enzymatic protein in PI3K/AKT signaling pathway, was found to be targeted by miR-301a [79] and miR-429 [88] in breast cancer and HCC respectively. It was shown that up-regulated miR-301a accelerates breast cancer progression by targeting PTEN, consequently resulting in Wnt signaling activation. The study suggests that miR-301a may be a potential therapeutic target for breast cancer. MiR-429 increases the metastatic capability of HCC by activating both Wnt and PI3K signaling through PTEN, suggesting miR-429 as a novel target for HCC treatment.

Enhancer of zeste homolog 2 (EZH2) catalyzes the methylation of histone H3 in target gene promoters to repress gene expression. It regulates cell proliferation, migration, metastasis, and chemo-resistance by silencing tumor suppressor genes such as APC [89–91]. EZH2 overexpression leads to activation of the Wnt pathway *via* APC reduction. EZH2 was reported to be targeted by miR-506 [92] and miR-144 [93] in colon and bladder cancer respectively. MiR-506 abrogates tumor proliferation and metastasis in colon cancer and miR-144 decreases cell growth in bladder cancer *via* Wnt pathway inhibition.

In another example, ZNRF3, an E3 ubiquitin ligase, inhibits Wnt pathway by increasing proteolysis of FZD and LRP6 [94]. It was reported that miR-146b-5p induces EMT and may promote papillary thyroid cancer metastasis through Wnt pathway activation by targeting ZNRF3. MiR-93 was also found to target ZNRF3 to promote lung carcinoma growth through Wnt signaling activation [95]. These data suggest ZNRF3 as a potential therapeutic target for thyroid and lung cancer.

#### Targeting receptor associated proteins in other signaling pathways

In addition to transcriptional factors and enzymes, miRs also regulate the Wnt pathway by targeting receptor associated proteins. For instance, ROBO receptors are members of the immunoglobulin superfamily of cell adhesion molecules (ICAMs). The ROBO pathway has been implicated in cancer development and progression [96]. The molecular mechanism by which dysregulated ROBO signaling promotes tumor progression is not well studied. ROBO3 was identified as a target of miR-383. ROBO3 increases Wnt pathway activity by sequestering sFRP to augment pancreatic cancer progression [81].

SLC34A2 is a plasma membrane protein that mediates sodium-dependent phosphate transport. SLC34A2 is involved in tumorigenesis. MiR-410 promotes the tumorigenesis and development of NSCLC by down-regulating SLC34A2 and activating the Wnt pathway. MiR-410 might be a new potential therapeutic target for NSCLC [82].

Integrin beta-like 1 (ITGBL1), is a beta-integrin similar protein. The function of ITGBL1 is not clear to date. ITGBL1, targeted by miR-576-5p, inhibits NSCLC progression by suppressing the Wnt pathway [97].

Additional examples of miRs that regulate Wnt by targeting elements in other signaling pathways are listed in Table 3. Wnt/β-catenin and other signaling pathways converge at numerous nodes of the cellular regulatory network, therefore, miRs regulate the Wnt pathway indirectly by targeting the node proteins with differential functions. Most of the targeted proteins are emerging novel oncogenes or tumor suppressors thereby the specific mechanisms by which they modulate Wnt/β-catenin signaling are not well understood. Additional studies revealing the regulation mechanisms will provide novel insight into the complex regulatory network that leads to tumorigenesis, cancer metastasis, and drug-resistance.

### The Wnt pathway regulates the expression of miRs

Thus far, it has been shown that miRs regulate the Wnt pathway however, the reverse is true-Wnt pathway modulates the expression of miR positively and negatively in a variety of tumors to induce carcinogenesis, progression, and drug resistance (Table 4). The aberrant expression profile of miRs was in part due to the constitutive activation of Wnt pathway in a plethora of cancers. β-catenin enhances downstream target expression by binding to TCF in the promoter and recruiting a panel of co-activators to trans-activate gene transcription.

**Table 4:** MiRs up or down regulated by Wnt signaling

Cancer Type	Down	Up	Reference
CRC	miR-215,miR-137	miR-708, miR-31, miR-135b, miR-21, miR-145, miR-126, miR-139-5p, miR-574-3p, miR-30e, miR-150	[98, 100, 101, 161]
Gastric cancer	miR-1234-3p, miR-135b-5p, miR-210,miR-4739,miR-122a	miR-20a-3p, miR-23b-5p, miR-335-3p, miR-423-5p,miR-455-3p	[103, 104]
HCC	miR-375	miR-770, miR-183/96/182	[106, 108]
Breast cancer	let-7	miR-182, miR-125b	[109–111]

In colon cancer, Brian et al. have used quantitative PCR arrays as well as mathematical analysis to discover distinctively expressed miRs in tumors resulting from loss of function mutation of APC. MiR-215 and -137 are repressed and miR-708, -135b and -31 levels are increased in APC mutation induced tumors. Target prediction and pathway analysis suggest that these miRs control signaling pathways critical for transformation [98]. Conversely, the expression profile of miRs was explored in TCF activity disrupted cells [99]. The miR transcriptome was assessed with the TaqMan Array in DLD1 CRC cells expressing dominant negative (dn) TCF4 alleles. Fifty-one miRs were found to be upregulated and 9 downregulated by at least two fold in dnTCF4 cells. Sixteen of the increased miRNAs, such as miR-574-3p,-139-5p and -30e-3p, were shown to be markedly reduced in CRC tissue. Some selected miRs (miR-30e-3p -145, -139-5p and -126) significantly suppress cell proliferation. Consistently, miR-30e is trans-activated by β-catenin/TCF4 complex during intestinal cell differentiation in rat [100]. In another study, a bioinformatics approach with ChIP-PCR was utilized to discover that TCF4 trans-activates miR-21 by directly binding to its promoter in colon cancer cells [101]. Similarly, miR-21 is trans-activated by β-catenin in a STAT3 dependent manner to promote cell invasion in glioma [102]. Aberrant Wnt/β-catenin signaling is the driving force for CRC carcinogenesis and progression. These identified downstream miRs of the Wnt pathway reveal part of the mechanisms through which Wnt/β-catenin promotes CRC tumorigenesis.

Other than CRC, 30% of gastric cancers carry nuclear accumulation of β-catenin, highlighting the importance of the Wnt pathway in gastric oncogenesis. To identify the miRs regulated by Wnt signaling, a miR microarray was conducted with β-catenin interference in gastric cancer cells to detect expression differences in the miR transcriptome [103]. The expression of miRs-4739, -210, -135b-5p, and-1234-3p are significantly increased and that of miRs-23b-5p, -20a-3p, -423-5p, -455-3p and -335-3p are significantly decreased. β-catenin interference results in delayed cell proliferation, increased apoptosis, weakened invasion of gastric cancer cells, and increased chemo-sensitivity of cancer cells. Also in gastric cancer, Wang et al. have demonstrated that miR-122a, a new tumor suppressor, is down-regulated by Wnt/β-catenin signaling [104]. The down-regulation of miR-122a mediated by aberrant Wnt pathwaysignaling is critical for the pathogenesis of gastrointestinal cancer.

Additionally, several studies explored the underlying mechanisms of HCC progression from the perspective of Wnt signaling, as β-catenin is a major oncogene in HCC and is activated in 30-40% of cases [105]. Wnt/β-catenin signaling was shown to up-regulate miR-770 to promote cell proliferation in HCC [106]. Also in HCC, Wnt signaling elevates miR-183/96/182 levels to augment cell invasion. β-catenin was found to increase the miR-183/96/182 transcription by physically interacting with TCF complex on miR-183/96/182 promoter region [107]. Another study found a strong correlation between β-catenin-activating mutation and down-regulation of miR-375 expression in HCC according to miR profiling in hepatocellular tumors and normal liver samples [108].

In breast cancer, miR-182 is highly expressed and is up-regulated by the Wnt pathway. The overexpression of miR-182 increases tumorigenicity and invasiveness by repressing RECK [109]. In addition, the expression of miR-125b is elevated by overexpression of SNAIL in a β-catenin dependent manner to enhance cancer stem cell enrichment and chemo-resistance in breast cancer [110]. This suggests that miR-125 may serve as a novel target to overcome chemo-resistance in cancer cells. Lastly, let-7 miR was discovered to be repressed by Wnt pathway *via* trans-activating Lin28 in breast cancer stem cells [111]. It was demonstrated that the Wnt pathway induces Lin28 up-regulation and let-7 down-regulation to enhance breast cancer cell expansion.

### Feedback loop between miRs and the Wnt pathway

There are positive feedback circuits between miR and Wnt signaling. MiR-218 and Wnt pathway form a positive feedback loop to increase osteoblast differentiation and abnormal expression of osteoblastic genes in breast cancer cells [112]. MiR-218 activates the Wnt pathway by targeting three Wnt signaling inhibitors (Sclerostin (SOST), DKK2, and sFRP2) during the process of osteogenesis. MiR-218 is also induced by active Wnt signaling, creating a positive feedback loop. MiR-146a expression is activated by SNAIL in a β-catenin dependent manner in CRC stem cells. In turn, miR-146a stabilizes β-catenin by targeting Numb [113]. A feedback loop is formed to direct symmetric cell division through activation of the Wnt pathway. MiR-372&373 induced by Wnt dependent transcription in turn, activates Wnt/β-catenin signaling by targeting Wnt signaling inhibitors including DKK1 [36].

Mutual inhibition between miR and Wnt pathway has also been observed. In glioma cells, miR-122 inhibits the Wnt pathway, which negatively regulates the expression of miR-122 [114]. In CRC, miR-101 and the Wnt pathway form a mutual inhibitory relationship [51]. The Wnt pathway activity represses the expression of miR-101 while overexpression of miR-101 strongly impairs β-catenin nuclear accumulation.

In addition, a negative feedback loop was also found between miR and the Wnt signaling pathway. MiR-483-3p targets β-catenin which induces the expression of miR-483-3p [115]. They form a negative feedback circuit in normal cells however, this negative feedback loop is inactivated if β-catenin carries an activating mutation.

In summary, miRs regulate the Wnt signaling pathway and Wnt signaling in turn modulates expression of miRs in human neoplasms. In addition, both form mutual feedback circuits, thereby increasing the connectivity and complexity of the regulatory network. Chartering this network will facilitate the development and advancement of miR-based clinical applications.

## MIR-BASED THERAPEUTICS

Recently, miR-based therapeutics have been developed based on the regulatory network of miRs and Wnt/β-catenin. MiR-based anticancer therapeutic approaches have used several strategies such as miR sponges, anti-miR oligonucleotides, miR masks, and small molecule inhibitors (reviewed in [1]). MiR-34 is lost or down-regulated in different types of cancer such as glioblastoma, HCC, cervical, ovarian, colon, and lung cancer. The ability of miR-34 to regulate a set of multiple Wnt signaling components in WNT pathway (discussed above) makes it an excellent candidate for novel Wnt-targeted therapy. MRX34 is a liposomal mimic of miR-34. A multicenter Phase I clinical trial of MRX34 is currently recruiting patients (ClinicalTrials.gov; NCT01829971). MRX34 is used for patients with advanced HCC, other selected solid cancers and hematologic malignancies. The scope of the trial is to establish the safety, pharmacokinetics and pharmacodynamics of MRX34. The company planned to finish the phase 1 trial by the end of 2016 and start the phase 2 clinical trial in 2017.

Pioneering work has certain challenges that must be overcome before miR-based therapies can be used in a clinical setting. Low bioavailability, specific tissue delivery, off target side effects, miR instability, immunogenicity and tumorigenicity are major problems to solve before the application in human clinical trials [1]. Despite these challenges, targeting miRs in cancer to rewire signaling networks is an applicable and rational strategy with great potential for success.

## CONCLUSIONS

Collectively, we have summarized the current understanding of crosstalk between miRs and the canonical Wnt signaling cascade in various types of tumors. Communication intersects in multiple planes to modulate cell proliferation, migration, cancer metastasis, and drug response. MiR-based therapeutics have entered clinical trials; a stage where opportunities and challenges co-exist for miR-based therapeutics for cancer. A better understanding of miRs and the Wnt signaling regulatory network will provide insights to further the development of miR-based remedies.
